# Supplementary material for: A phase 2 trial of burosumab for treatment of fibroblast growth factor-23-mediated hypophosphatemia in children and adults with fibrous dysplasia
Source: Bone Res. 2026 Apr 27;14:47. doi: 10.1038/s41413-026-00523-7 (PMC13111699; doi:10.1038/s41413-026-00523-7)
Supplement: Supplementary file 1 — Supplementary Appendix [file 41413_2026_523_MOESM1_ESM.docx]

# Supplementary Appendix:

# A phase 2 trial of burosumab for treatment of fibroblast growth factor-23 mediated hypophosphatemia in children and adults with fibrous dysplasia

Contents

[Supplementary Appendix: 1](#_Toc216427215)

[A phase 2 trial of burosumab for treatment of fibroblast growth factor-23 mediated hypophosphatemia in children and adults with fibrous dysplasia 1](#_Toc216427216)

[Figure S1. Overall trial design 2](#_Toc216427217)

[Figure S2. Clinical flowchart 3](#_Toc216427218)

[Table S1. Individual participant characteristics 4](#_Toc216427219)

[Figure S3. Bone turnover markers 5](#_Toc216427220)

[5](#_Toc216427221)

[5](#_Toc216427222)

[Figure S4. Additional Patient-Reported Outcomes 6](#_Toc216427223)

[Table S5. Activities of Daily Living Questionnaire 7](#_Toc216427224)

[Figure S5. Safety labs 8](#_Toc216427225)

[Figure S6. Markers of lesion activity 9](#_Toc216427226)

[Figure S7. H&E stains of biopsies before and after burosumab treatment 10](#_Toc216427227)

[Figure S8. Phosphate and alkaline phosphatase levels before and after burosumab treatment. 11](#_Toc216427228)

[Table S9. Detailed summary of all hyperphosphatemic episodes 12](#_Toc216427229)

## Figure S1. Overall trial design


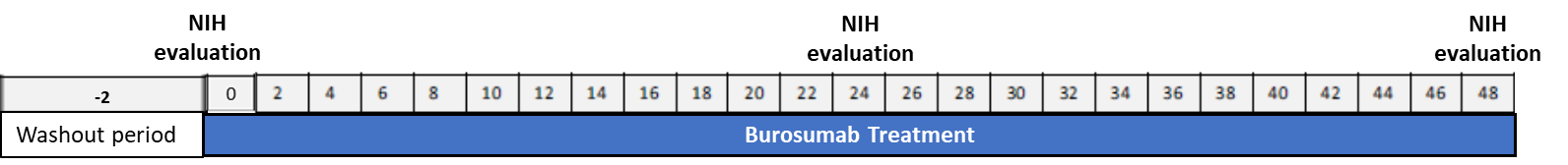


Participants were treated with burosumab for 48 weeks following a 2-week washout period. Evaluations were performed at the NIH Clinical Center at baseline, 24, and 48 weeks. Between visits burosumab was administered locally, and participants were monitored remotely with outpatient labs.

## Figure S2. Clinical flowchart


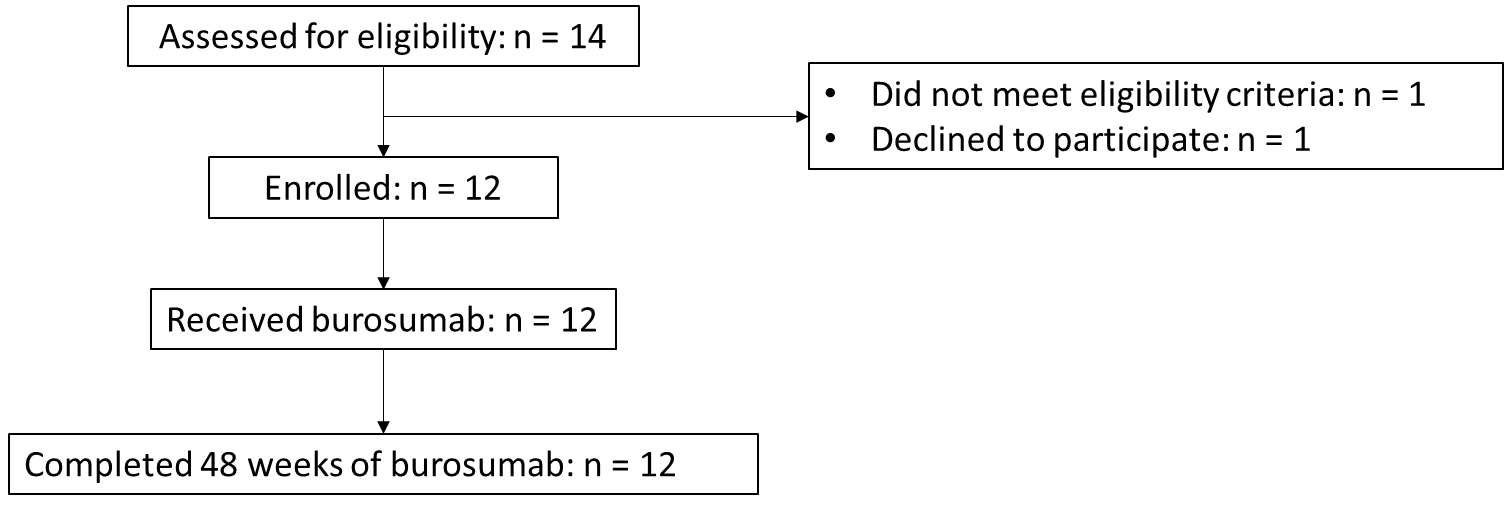


## Table S1. Individual participant characteristics

| **ID** | **Age (years) & Sex** | **Skeletal Burden Score^1^** | **MAS-associated endocrinopathies** | **Concomitant anti-resorptive treatment** | **Previous conventional therapy** | **Burosumab regimen at study completion** |
| --- | --- | --- | --- | --- | --- | --- |
| BUR01 | 38F | 68 | Hypercortisolism (adult-onset, resolving^2^), precocious puberty, hyperthyroidism, growth hormone excess | None | Yes | 0.7 mg/kg every 2 weeks |
| BUR02 | 25F | 75 | Precocious puberty | Pamidronate^4^ | Yes | 0.7 mg/kg every 2 weeks |
| BUR03 | 13F | 61 | Precocious puberty, hyperthyroidism, growth hormone excess | None | Yes | 0.8 mg/kg every 2 weeks |
| BUR04 | 6F | 75 | Precocious puberty, hyperthyroidism, growth hormone excess, hypercortisolism (neonatal-onset, resolved^3^) | None | Yes | 2.3 mg/kg every 2 weeks |
| BUR05 | 12F | 35 | Precocious puberty | None | Yes | 0.5 mg/kg every 2 weeks |
| BUR06 | 23F | 43 | Precocious puberty | Denosumab^5^ | Yes | 1.0 mg/kg every 2 weeks |
| BUR07 | 16M | 75 | Precocious puberty, hyperthyroidism, growth hormone excess | None | Yes | 0.8 mg/kg every 2 weeks |
| BUR08 | 10M | 51 | Hyperthyroidism, growth hormone excess | None | Yes | 0.4 mg/kg every 2 weeks |
| BUR09 | 8M | 69 | None | None | Yes | 1.5 mg/kg every 2 weeks |
| BUR10 | 33F | 69 | Precocious puberty | Denosumab^6^ | Yes | 0.5 mg/kg every 2 weeks |
| BUR11 | 36F | 28 | Precocious puberty | Denosumab^6^ | Yes | 0.6 mg/kg every 4 weeks |
| BUR12 | 16M | 48 | Precocious puberty, growth hormone excess | None | No | 0.3 mg/kg every 2 weeks |

^1^Semi-quantitative measure of the proportion of the skeleton involved with FD, ranging from 0 (no FD) to 75 (panostotic FD). See Collins et al, J Bone Min Res 2005 for details. ^2^Participant was diagnosed with hypercortisolism prior to burosumab initiation and underwent bilateral adrenalectomy at week 7. ^3^Participant underwent bilateral adrenalectomy in infancy. ^4^Participant received pamidronate 30 mg every 3 months as needed for treatment of FD-bone pain for the prior 10 years. She was administered one dose at week 12, and did not require any further doses over the course of the trial. ^5^Particiant received denosumab 120 mg every 3 months for the previous 1 year for treatment of FD-related bone pain and disability and continued this dose throughout the trial. ^6^Participant received denosumab 60 mg every 3 months for the previous 2 years for treatment of FD-related bone pain and disability and continued this dose throughout the trial.

## Figure S3. Bone turnover markers

##
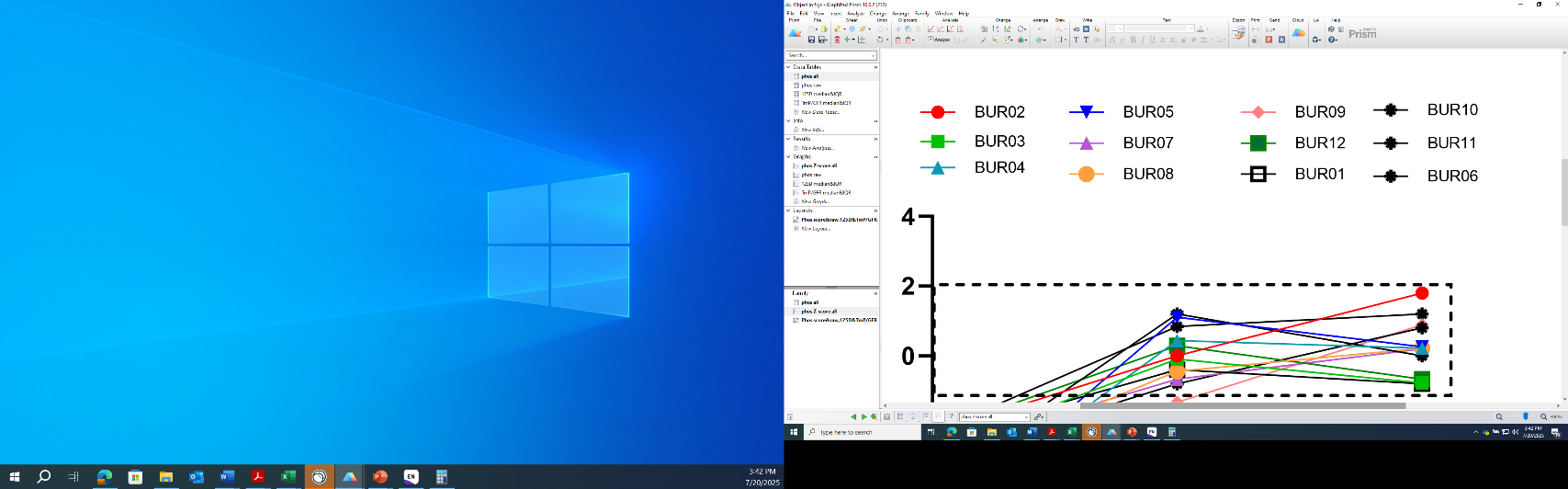


##

Individualized changes in procollagen-1 propeptide (A) and C-terminal telopeptides (C) in the entire cohort (n=12). Note that participants shown in black have confounding variables impacting bone turnover markers, including concomitant denosumab treatment (BUR10, 11, and 06, stars), and hypercortisolism status-post total adrenalectomy at week 7 (BUR01, open box). Percent change from baseline in procollagen-1 propeptide (B) and C-terminal telopeptides (D) in the remaining cohort (n=8) are shown as median and interquartile range.

## Figure S4. Additional Patient-Reported Outcomes


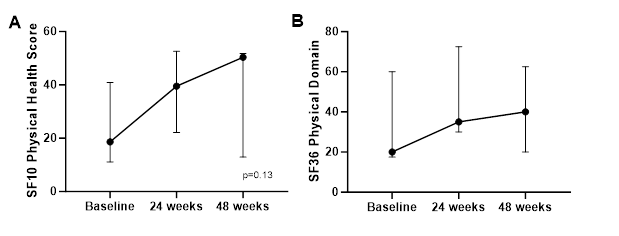


Data are expressed as median and interquartile range. Panel A shows a potential trend toward improvement in SF10 physical health scores in pediatric participants (p=0.13, signed rank test, n=7). Panel B showed results for SF36 physical domain scores in adult participants, with no clear trend (n=5).

## Table S5. Activities of Daily Living Questionnaire

| Complete items 1-3 at Baseline Visit | | Indicate level of change since Baseline on extent of impact | |
| --- | --- | --- | --- |
|  |  | Complete items 4-6 at Week 24 | Complete items 7-9 at Week 48 |
| Please list up to 3 activities of daily living that are affected by FD/skeletal features at home or school/work (e.g., mobility, climbing stairs, dressing, playing with peers, catching a bus); and estimate the extent of impact | 1) Activity:  Stops activity completely  Extremely limited  Moderately limited  Slightly limited | 4) Since last visit  Very Much Improved  Much Improved  Minimally Improved  No Change  Minimally Worse  Much Worse  Very Much Worse | 7) Since baseline  Very Much Improved  Much Improved  Minimally Improved  No Change  Minimally Worse  Much Worse  Very Much Worse |
|  | 2) Activity:  Stops activity completely  Extremely limited  Moderately limited  Slightly limited | 5) Since last visit  Very Much Improved  Much Improved  Minimally Improved  No Change  Minimally Worse  Much Worse  Very Much Worse | 8) Since baseline  Very Much Improved  Much Improved  Minimally Improved  No Change  Minimally Worse  Much Worse  Very Much Worse |
|  |  | N/A No activity listed in 2 | N/A No activity listed in 2 |
|  | 3) Activity: | 6) Since last visit | 9) Since baseline |
|  | Stops activity completely  Extremely limited  Moderately limited  Slightly limited | Very Much Improved  Much Improved  Minimally Improved  No Change  Minimally Worse  Much Worse  Very Much Worse | Very Much Improved  Much Improved  Minimally Improved  No Change  Minimally Worse  Much Worse  Very Much Worse |
|  |  | N/A: No activity listed in 3 | N/A: No activity listed in 3 |

## Figure S5. Safety labs

Data are presented as median and interquartile range.

##

## Figure S6. Markers of lesion activity

^18^F-NaF PET/CT metrics total activity (A) and SUV mean (B) are shown in median and interquartile range for the entire cohort (n=12). Histologic evaluation of pre- and post-treatment biopsies 4 adult participants (BUR02, 06, 10, and 11) show no quantitative differences in the cellularity (C) or proportion of fibrous tissue (D).

## Figure S7. H&E stains of biopsies before and after burosumab treatment


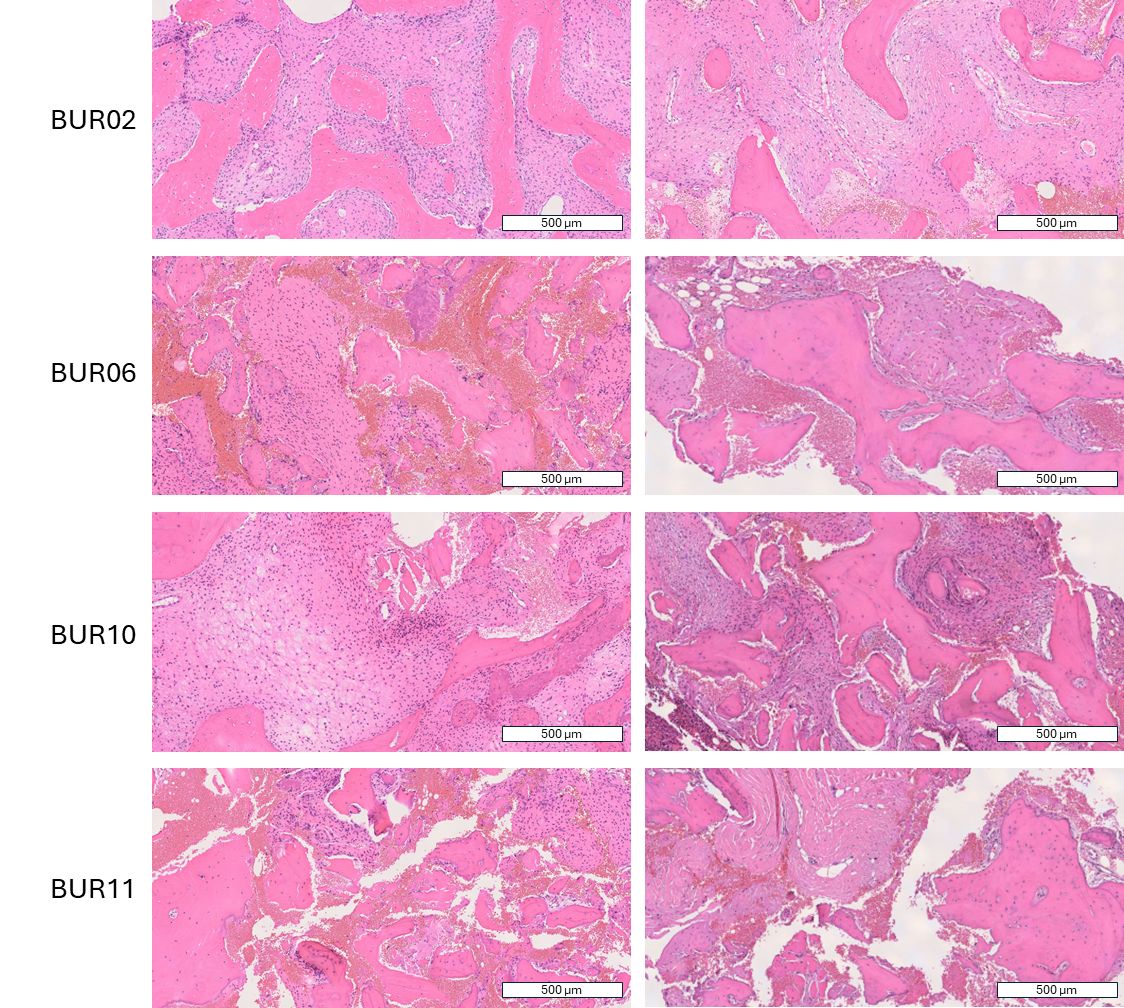


Low magnification images of a cross-section of all biopsies obtained from participants at baseline and 48 weeks. Locations included iliac crest (BUR02, 06, 10) and rib (BUR11).

## Figure S8. Phosphate and alkaline phosphatase levels before and after burosumab treatment.

Retrospective review of phosphate (A) and alkaline phosphatase levels (B) available prior to burosumab treatment. Participants who received treatment with phosphate supplements and active vitamin D analogs are shown in dashed lines.

## Table S9. Detailed summary of all hyperphosphatemic episodes

| **Participant** | **Week** | **Phosphate Z-score** | **Phosphate level (mg/dL)** | **Response** |
| --- | --- | --- | --- | --- |
| BUR05 | 4 | 2.9 | 6.3 | Dose #3 skipped. Repeat labs at week 6 showed hypophosphatemia with Z-score -3.6. Burosumab re-initiated at a reduced dose (0.6 mg/kg from 0.8 mg/kg every 2 weeks) |
| BUR08 | 2 | 2.4 | 6.1 | Dose #2 skipped. Repeat labs at week 4 showed normophosphatemia with Z-score -0.9. Burosumab re-initiated at a reduced dose (0.3 mg/kg from 0.8 mg/kg every 2 weeks) |
| BUR08 | 36 | 2.4 | 6.1 | Dose #18 skipped. Repeat labs at week 40 showed normophosphatemia with Z-score -0.7. Burosumab re-initiated at a reduced dose (0.4 mg/kg from 0.5 mg/kg every 2 weeks) |
| BUR09 | 2 | 2.4 | 6.1 | Dose #2 skipped. Repeat labs at week 4 showed hypophosphatemia with Z-score -2. Burosumab re-initiated at a reduced dose (0.6 mg/kg from 0.8 mg/kg every 2 weeks) |
| BUR10 | 36 | 3.4 | 5.2 | Dose #13 skipped. Repeat labs at week 40 showed normophosphatemia with Z-score 0.8. Burosumab re-initiated at a reduced dose (0.5 mg/kg from 0.6 mg/kg every 2 weeks) |
| BUR11 | 10 | 6 | 6.5 | Participant was receiving every 4 week dosing, with next dose (#4) due at week 12. Pre-dose labs at week 12 showed phosphate at the bottom of the target range with Z-score -1. Burosumab was continued at its current dose (0.5 mg/kg every 4 weeks) with continued monitoring |
| BUR11 | 32 | 3.2 | 5.1 | Dose #11 skipped. Repeat labs at week 36 showed normophosphatemia with Z-score -0.6. Burosumab re-initiated at a reduced dose (0.5 mg/kg every 4 weeks from 0.5 mg/kg every 2 weeks) |
| BUR12 | 2 | 5.0 | 6.6 | Dose #2 skipped. Repeat labs at week 4 showed phosphate just below the target range with Z-score -1.05. Burosumab re-initiated at a reduced dose (0.3 mg/kg from 0.8 mg/kg every 2 weeks) |
